# The Practitioners' Bookshelf

**Published:** 1893-03-25

**Authors:** 


### THE PRACTITIONERS' BOOKSHELF.

### ORTHOPÆDY.

Mr. Heather Bigg, the well-known specialist and writer on orthopædic surgery, is bringing out in five parts a manual of orthopædy,* the first of which, dealing with deformities and deficiencies of the head and neck, lies before us, the subsequent parts being promised at half-yearly intervals. As the manual contains only the outlines of a larger book, which the author tells us in his prefatory remarks he has been engaged on for some years, we hope that the scanty remarks on certain points in the present issue will be elucidated in more detail at a subsequent period. Though brief, the excellence of the book will more than compensate for the somewhat florid style which the author indulges in, for it gives an outline of the great advances made in relieving and curing the deformities of the head and neck. In it will be found many excellent methods of treatment suggested by the large experience which Mr. Heather Bigg has had in the branch of surgery he has made himself such a master of. Orthopædy, like many other sciences, has spread itself over a much wider field than was originally intended, for we are told that in the subsequent parts hernia and other structural debilities will be included. Mr. Heather Bigg seems to have an hereditary genius for inventing appliances and splints to relieve deformities. There is no index.

* "A Short Manual of Orthopædy. The Head and Neck." 73 pages. Heather Bigg, F.R.C.S. Edin. (J. and A. Churchill, 1892.)

